# Improved detection of *Mycobacterium tuberculosis* and *M. bovis* in African wildlife samples using cationic peptide decontamination and mycobacterial culture supplementation

**DOI:** 10.1177/10406387211044192

**Published:** 2021-09-11

**Authors:** Wynand J. Goosen, Léanie Kleynhans, Tanya J. Kerr, Paul D. van Helden, Peter Buss, Robin M. Warren, Michele A. Miller

**Affiliations:** DSI-NRF Centre of Excellence for Biomedical Tuberculosis Research, South African Medical Research Council Centre for Tuberculosis Research, Division of Molecular Biology and Human Genetics, Faculty of Medicine and Health Sciences, Stellenbosch University, Cape Town, South Africa; DSI-NRF Centre of Excellence for Biomedical Tuberculosis Research, South African Medical Research Council Centre for Tuberculosis Research, Division of Molecular Biology and Human Genetics, Faculty of Medicine and Health Sciences, Stellenbosch University, Cape Town, South Africa; DSI-NRF Centre of Excellence for Biomedical Tuberculosis Research, South African Medical Research Council Centre for Tuberculosis Research, Division of Molecular Biology and Human Genetics, Faculty of Medicine and Health Sciences, Stellenbosch University, Cape Town, South Africa; DSI-NRF Centre of Excellence for Biomedical Tuberculosis Research, South African Medical Research Council Centre for Tuberculosis Research, Division of Molecular Biology and Human Genetics, Faculty of Medicine and Health Sciences, Stellenbosch University, Cape Town, South Africa; Veterinary Wildlife Services, Kruger National Park, South African National Parks, Skukuza, South Africa; DSI-NRF Centre of Excellence for Biomedical Tuberculosis Research, South African Medical Research Council Centre for Tuberculosis Research, Division of Molecular Biology and Human Genetics, Faculty of Medicine and Health Sciences, Stellenbosch University, Cape Town, South Africa; DSI-NRF Centre of Excellence for Biomedical Tuberculosis Research, South African Medical Research Council Centre for Tuberculosis Research, Division of Molecular Biology and Human Genetics, Faculty of Medicine and Health Sciences, Stellenbosch University, Cape Town, South Africa

**Keywords:** African wildlife, culture, *Mycobacterium tuberculosis* complex, *Mycobacterium bovis*, South Africa, TiKa

## Abstract

In South Africa, mycobacterial culture is regarded as the gold standard for the detection of *Mycobacterium tuberculosis* complex (MTBC) infection in wildlife even though it is regarded as “imperfect.” We compared a novel decontamination and mycobacterial culture technique (TiKa) to the conventional mycobacterium growth indicator tube (MGIT) system using known amounts of bacilli and clinical samples from MTBC-infected African buffaloes (*Syncerus caffer*), white rhinoceros (*Ceratotherium simum*), and African elephants (*Loxodonta africana*). Use of the TiKa-KiC decontamination agent on samples spiked with 10,000 to 10 colony forming units (cfu) of *M. bovis* (SB0121) and *M. tuberculosis* (H37Rv) had no effect on isolate recovery in culture. In contrast, decontamination with MGIT MycoPrep resulted in no growth of *M. bovis* samples at concentrations < 1,000 cfu and *M. tuberculosis* samples < 100 cfu. Subsequently, we used the TiKa system with stored clinical samples (various lymphatic tissues) collected from wildlife and paucibacillary bronchoalveolar lavage fluid, trunk washes, and endotracheal tube washes from 3 species with known MTBC infections. Overall, MTBC recovery by culture was improved significantly (*p* < 0.01) by using TiKa compared to conventional MGIT, with 54 of 57 positive specimens versus 25 of 57 positive specimens, respectively. The TiKa mycobacterial growth system appears to significantly enhance the recovery of MTBC members from tissue and paucibacillary respiratory samples collected from African buffaloes, African elephants, and white rhinoceros. Moreover, the TiKa system may improve success of MTBC culture from various sample types previously deemed unculturable from other species.

Bovine tuberculosis (bTB) is caused by infection with *Mycobacterium bovis*; human tuberculosis (TB) is caused primarily by *M. tuberculosis*; both species are members of the *M. tuberculosis* complex (MTBC).^14^ Both bTB and TB are typically chronic diseases that are significant global health threats to human and animal populations.^18^ The presence of bTB in domestic livestock and wildlife can result in economic losses for cattle farmers and wildlife ranchers, and restricts wildlife conservation activities.^26,27^ These losses can be devastating to a country’s economy, especially those heavily dependent on animal-related industries such as agriculture and tourism.^14^ Reports of bTB in people from various African countries,^12,20,21^ and TB in domestic cattle, wildlife, and pets,^5,11,17,23^ demonstrate that these pathogens can be transmitted to a wide variety of susceptible hosts. Increased opportunities for disease transmission at human–animal interfaces occur as human settlements encroach on agricultural lands or natural wildlife habitats. Although cattle are known as the primary host in South Africa, *M. bovis* has been detected in numerous wildlife species including African buffaloes (*Syncerus caffer*) and rhinoceros (*Ceratotherium simum*, *Diceros bicornis*).^17,22^ *M. tuberculosis* has also been detected in a free-ranging African elephant bull in Kruger National Park (KNP; South Africa).^18^ These findings highlight the current transmission risk at the human–livestock–wildlife interface and the need for optimal detection tools.

Unfortunately, there is still a paucity of detection tests and disease surveillance programs for wildlife, and MTBC infections can remain undetected for years, resulting in uncontrolled spread.^4,10^ In cattle and buffaloes, the conventional antemortem diagnosis of bTB is based on the measurement of antigen-specific cell-mediated immune (CMI) responses, and the definitive diagnosis is based on detection of bacilli by mycobacterial culture.^9,15^ However, species-specific in vitro assays for detection of CMI responses to MTBC are either very limited, as in the case of African rhinoceros,^4^ or do not yet exist, as for African elephants. Therefore, antemortem TB diagnosis in these species relies primarily on mycobacterial culture of respiratory tract samples such as bronchoalveolar lavage fluid (BALF) from rhinoceros or BALF and trunk washes (TWs) from African elephants.

Mycobacterial culture is regarded as the “imperfect” gold standard for wildlife testing.^13,25^ This may be because of the slow growth of MTBC members coupled with the aggressive decontamination methods applied to polymicrobial field samples containing low numbers of pathogenic mycobacteria.^1,19^ To overcome these challenges with paucibacillary samples, a next-generation mycobacterial culture method, the TiKa system (TiKa Diagnostics), was developed by improving several features of the conventionally used mycobacterial growth indicator tubes (Bactec MGIT; Becton Dickinson).^3^ These improvements include the substitution of the conventional decontamination procedure with a cocktail of highly active antimicrobial short cationic D-enantiomer peptides, selected for their lack of antimicrobial activity against mycobacteria (TiKa-KiC), in conjunction with another cationic D-enantiomer (supplement B) that stimulates mycobacterial growth. Only one study using this method in animal samples has been published, in which *M. avium* subsp. *paratuberculosis* (MAP) was cultured from cattle specimens.^3^ That study showed similar diagnostic sensitivity of the TiKa system to that of a quantitative PCR (qPCR), with increased detection of non-replicating MAP in lymph node tissues, even those MAP that remained undetected by the conventional MGIT system.^3^

We compared the effect of decontamination procedures and addition of a novel growth-enhancing peptide on mycobacterial growth in the TiKa and conventional MGIT culture systems. We further describe here the use of the TiKa system for the detection of *M. tuberculosis* and *M. bovis* from stored clinical tissue and paucibacillary respiratory tract samples from 3 wildlife species with known MTBC infection status.

## Materials and methods

### Preculture treatment and mycobacterial culture of *M. bovis* and *M. tuberculosis*

*M. bovis* SB0121 and *M. tuberculosis* H37Rv stock cultures (Stellenbosch University) were subcultured in separate T25 culture flasks (Thermo Fisher) containing 5 mL of liquid Middlebrook 7H9 broth medium (Merck), 0.05% Tween 80 (Merck), 0.2% glycerol, and 10% Middlebrook oleic acid dextrose citrate (OADC) growth supplement (Merck) at 37°C for 5 d. The optical density (OD) was determined, and cultures diluted to an OD = 0.05 in 30 mL of 7H9 culture medium in T75 culture flasks. The cultures were subsequently incubated for 2 d until they reached an OD = 0.2. Serial dilutions (10^−1^–10^−6^) were prepared in round-bottom 48-well culture plates. The neat culture and 3 dilutions (10^−4^–10^−6^) were plated in triplicate on vented petri dishes containing 7H11 Middlebrook agar (Merck), 10% OADC, and 5% glycerol. Colony forming units (cfu) were enumerated after 30 d, and the final bacilli amount (expressed as cfu) was determined for all stored liquid cultures.

Before using the above-mentioned liquid cultures for our dilution series, all liquid cultures were first agitated by vortex for 15 s in tubes containing sterile zirconium silica beads (Merck) to disrupt clumps of bacteria. The single cell suspension was transferred to sterile microcentrifuge tubes and centrifuged at 3,000 × *g* for 20 min. The supernatant was discarded, and the pellet was resuspended in sterile PBS ready for immediate use.

Serial dilutions of specific working concentrations (cfu/mL) of *M. tuberculosis* and *M. bovis* were prepared in triplicate on the same day. Aliquots were serially diluted in a final volume of 1 mL of sterile PBS, in duplicate, to obtain the desired amount of bacilli before preculture decontamination treatment of each replicate dilution as a single event on the same day. Briefly, the replicate dilution samples of 10,000 to 10 cfu for *M. tuberculosis* and 10,000 to 10 cfu for *M. bovis*, were pretreated, in 3 different ways, prior to inoculation into MGIT tubes: 1) no decontamination (controls), 2) decontaminated with BBL MycoPrep (Becton Dickinson) containing acetyl-L-cysteine–sodium hydroxide (NALC-NaOH) as described by the manufacturer, and 3) decontaminated with TiKa-KiC (TiKa Diagnostics). TiKa-KiC decontamination requires specimen incubation in the KiC decontamination agent at 37℃ for 20 h prior to growth indicator tube inoculation. The pretreated duplicate dilution samples were inoculated into conventional MGIT tubes containing BBL MGIT PANTA-OADC enrichment media (Becton Dickinson) and TiKa tubes containing PANTA-OADC enrichment media and the TiKa supplement B reagent (TiKa Diagnostics). All tubes were transferred to the Bactec MGIT 960 mycobacterial detection system (Becton Dickinson) and monitored frequently as described previously.^2,9^ We defined the limit of detection (LOD) as the fewest cfu that, when spiked into 1 mL of PBS, would result in 100% detection of *M. tuberculosis* and *M. bovis* (within 56 d) by the mycobacterial culture system. Time to positivity (TTP) was defined as the time at which growth (using a growth index of 100 to define active growth) was detected within the MGIT tubes by the instrument from the time they were placed in the MGIT culture detection system.

### Wildlife sample selection

We selected pooled lymph node (mandibular, retropharyngeal, mediastinal, tracheobronchial) samples of 26 African buffaloes (pooled sample set per animal), known to be infected with *M. bovis* (confirmed by mycobacterial culture). Samples were collected between 2016 and 2018 as part of bTB test-and-cull programs from Hluhluwe iMfolozi Park (KwaZulu Natal province), a bTB endemic area in South Africa. All buffaloes were tested using the single comparative intradermal tuberculin test, 2 separate interferon-gamma (IFN-γ) release assays (IGRAs), and a QuantiFERON TB Gold In-tube IFN-γ induced protein of 10 kDa (IP-10) release assay. Test-positive (i.e., positive on any test) animals were culled and postmortem examination conducted. Tissue specimens were collected at autopsy for conventional mycobacterial culture.

We included 25 tissue samples from white rhinoceros with known *M. bovis* infection (confirmed by mycobacterial culture). These were collected opportunistically from 6 poached rhinoceros (~2–6 lymph node and lung specimens/animal) in KNP, that were sampled within 6 h of death.

Furthermore, we selected 3 tissue samples (1 lung specimen, 1 pool of head lymph nodes, 1 pool of thoracic lymph nodes) from 1 free-ranging and 1 zoo elephant with known *M. bovis* and *M. tuberculosis* infections, respectively. Additionally, we collected one TW, one BALF sample, and one endoscopic tube wash sample from the *M. tuberculosis*–infected zoo elephant prior to euthanasia.

Ethical approval for our project was granted by Stellenbosch University Animal Care and Use Committee (ACU-2018-0966, ACU-2019-6308, ACUACU-2019-9081) and section 20 research permits issued by the Department of Agriculture, Land Reform and Rural Development (DALRRD), formerly known as the Department of Agriculture, Forestry and Fisheries (ref: 12/11/1/7/2, 12/11/1/7/6). A biomaterial transfer agreement was also granted by the South African National Park (SANParks) Animal Care and Use Committee (ref: 011/19 and 05/11), and all animals were handled according to the SANParks Standard Operating Procedures for the Capture, Transportation and Maintenance in Holding Facilities of Wildlife.

### Sample collection and processing

All animal carcasses that we included were examined for gross TB lesions. Tissue samples with TB-consistent lesions were collected separately and stored at –20℃. For animals with no visible lesions, samples of head (mandibular, retropharyngeal) and thoracic (mediastinal, tracheobronchial) lymph nodes were pooled by anatomic site. Endoscopic bronchoalveolar lavages were performed on chemically immobilized rhinoceros and elephants, as described previously, to collect antemortem samples.^16,24^ Additionally, TW samples were obtained from immobilized elephants by flushing 500 mL of sterile saline into each nostril, separately, elevating and lowering the trunk for 30–40 s, and aspirating the fluid into a sterile 500-mL collection chamber. Respiratory samples collected in 50-mL sterile Falcon tubes were concentrated by centrifugation at 2,000 × *g* for 30 min and decanting 46 mL of the supernatant.^7,8^ The remaining 4 mL with resuspended pellet were stored at –20°C, transported to the laboratory, and processed for downstream mycobacterial culture.

Briefly, ~10 g of tissue were homogenized in 50-mL skirted tubes (Becton Dickinson) containing eight 4.8-mm metal beads and 4 mL of sterile PBS using a blender (Bullet Blender 50; Next Advance) for 15 min at maximum speed. All tissue homogenates and concentrated respiratory samples were processed in parallel (equal portions) for mycobacterial culture using: 1) the conventional MGIT, and 2) the TiKa mycobacterial culturing system.^3,9^ Both culturing approaches were performed using the Bactec MGIT 960 mycobacterial detection system (Becton Dickinson), in which TTP was recorded for all bacterial growth detected after 42 d in the MGIT tubes. Thereafter, all liquid cultures with detected bacterial growth were further subcultured onto blood agar plates to exclude any possible contaminations, and subjected to Ziehl–Neelsen (ZN) acid-fast staining for microscopic MTBC confirmation as described previously.^6^ All samples were subjected to MTBC confirmation by genetic speciation using the region of difference (RD) PCR test (RD-PCR), as described previously.^28^ All culture-positive specimens that stained ZN-positive, that yielded no growth on blood agar plates, and that were confirmed infected with MTBC by RD-PCR, were defined as “MTBC infected.”

### TiKa decontamination and culture

The TiKa-KiC sample decontamination step required 2 mL of tissue homogenate or 2 mL of concentrated respiratory samples. Prior to decontamination, samples were centrifuged at 14,000 × *g* for 10 min and the supernatant discarded. Thereafter, the pellet was resuspended in 10 mL of half-strength Mueller–Hinton broth (Merck) supplemented with TiKa-KiC and incubated for 20 h at 37℃ with gentle shaking. Samples were centrifuged at 1,600 × *g* for 30 min and the pellet resuspended in 500 µL of sterile PBS. TiKa liquid cultures used 7-mL MGIT tubes containing 800 µL of antimicrobial growth supplement mixture (PANTA + OADC; Becton Dickinson), 8.5 µL of growth supplement B (TiKa Diagnostics), and 500 µL of each treated sample.^3^ Equal amounts of tissue homogenates and respiratory samples were used for both culture systems.

### Statistical analysis

The median TTP in days and hours for the detection of mycobacterial growth by the Bactec MGIT 960 mycobacterial detection system was plotted as bar graphs, with y-axis as TTP values, x-axis as cfu, and interquartile ranges generated for all technical and biological replicates (Prism v.7; GraphPad). Continuous TTP results were summarized as median and interquartile ranges (IQR) in Excel (Microsoft). For each of the 4 spiked bacterial concentrations, differences between TTPs from each of the 6 treatment groups were compared using an omnibus repeated-measures ANOVA statistical test (Prism v.7). Subsequently, significance (*p* < 0.01) was confirmed between treatment groups with multiple *t*-tests using the Bonferroni–Dunn method to correct for multiple testing (alpha = 0.01).

MTBC recovery from clinical specimens was defined as the number of specimens with confirmed MTBC growth (confirmed by RD-PCR, positive ZN staining, negative blood agar growth) ÷ number of samples cultured from known MTBC-infected animals. The McNemar test was used to compare (*p* < 0.01) culture results from buffalo and rhinoceros clinical specimens using QuickCalcs software (https://www.graphpad.com/quickcalcs/McNemar1.cfm). Moreover, significance (*p* < 0.01) was confirmed between TTPs of each animal specimen cultured in parallel with both techniques using the Wilcoxon signed rank test for paired observations (Suppl. Tables 1, 2).

## Results

### Use of the TiKa and MGIT systems on pure cultures of MTBC isolates

**Figure 1.**
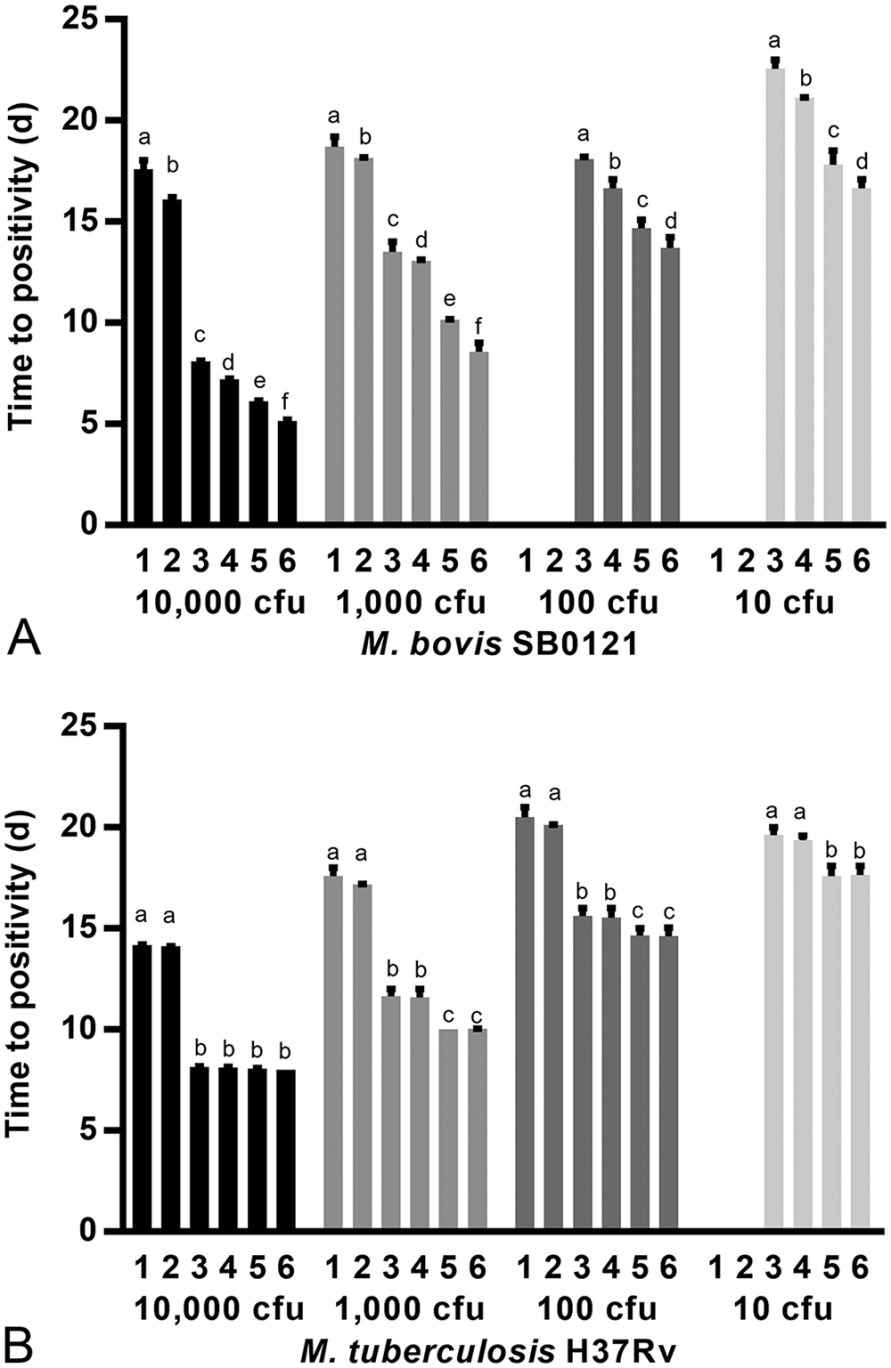
Conventional Bactec MGIT (MGIT tube containing PANTA-OADC antimicrobial enrichment media) and TiKa (MGIT tube containing PANTA-OADC and TiKa supplement B) culture results represented as median time to positivity (d) with interquartile ranges (IQR) for sterile phosphate buffer spiked in duplicate with known amounts (cfu) of **A.** *Mycobacterium bovis* SB0121 (10,000–10 cfu) and **B.** *M. tuberculosis* H37Rv (10,000–10 cfu), respectively. Prior to inoculation into growth indicator tubes, all spiked samples were either: decontaminated with MycoPrep (treatment 1: MycoPrep MGIT; treatment 2: MycoPrep TiKa), not decontaminated (treatment 3: MGIT control; treatment 4: TiKa control), or decontaminated with KiC agent (treatment 5: KiC MGIT; treatment 6: KiC TiKa). For some data points, the IQRs were shorter than the height of the bar; in these cases, the ranges were not drawn. Using the multiple *t*-test with Bonferroni–Dunn correction for multiple testing (alpha = 0.01), statistical significance (*p* < 0.01) was calculated between treatments for each amount of mycobacteria. Within each amount of the target *Mycobacterium*, bars with a different superscript are significantly different at *p* < 0.01.

After decontaminating serial dilutions of *M. bovis* (SB0121) and *M. tuberculosis* (H37Rv) cultures (dilutions prepared in PBS) with TiKa KiC, *M. bovis* and *M. tuberculosis* were successfully cultured from all dilutions from 10,000 to as low as 10 cfu (Fig. 1A, 1B). In contrast, following decontamination with MycoPrep containing NALC-NaOH, no growth was observed in *M. bovis* samples containing <1,000 cfu (Fig. 1A) and in *M. tuberculosis* samples <100 cfu (Fig. 1B). No growth was observed in the negative controls (0 cfu). Decontamination of samples with MycoPrep also resulted in a significantly (*p* < 0.01) longer TTP in samples in which *M. bovis* and *M. tuberculosis* could be detected, compared to either no decontamination in the controls or to TiKa-KiC peptide decontamination (Fig. 1A, 1B). Decontamination of samples with the TiKa-KiC agent resulted in significant (*p* < 0.01) reduction in the TTP compared to no decontamination for all *M. bovis* cfu and ≤1,000 *M. tuberculosis* cfu (Fig. 1A, 1B).The addition of TiKa supplement B reagent to TiKa cultures (Fig. 1A, 1B; treatments 2, 4, 6) significantly (*p* < 0.01) enhanced the growth of *M. bovis*, independent of decontamination treatment, but not the growth of *M. tuberculosis* (Fig. 1A, 1B).

### Comparison of conventional MGIT and TiKa systems for clinical specimens from MTBC-infected animals

Of the 26 tissues (pooled lymph nodes) collected from known MTBC-infected African buffaloes, a significantly (*p* = 0.0004) increased MTBC culture recovery rate (MTBC species confirmed by PCR in culture-positive specimens), from 10 of 26 to 23 of 26, was observed when using the TiKa system compared to the conventional MGIT system (Table 1). Additionally, a significant (*p* = 0.005) average overall reduction in TTP of 13.4 d for *M. bovis* growth in TiKa was observed. Differences between culture outcomes from the MGIT and TiKa systems were compared for all MTBC-infected buffaloes and was found to be significant using the McNemar test (*p* = 0.0059). Both systems correctly identified MTBC infections in 7 of 26 specimens, with 19 of 26 discrepancies (MGIT *M. bovis*–positive and TiKa false-negative = 6; MGIT false-negative and TiKa *M. bovis*–positive = 13; Suppl. Table 1).

**Table 1.** Comparative mycobacterial culture results for tissue and respiratory samples (bronchial alveolar lavage fluid and trunk washes) collected from 26 known *Mycobacterium tuberculosis* complex (MTBC)-infected African buffaloes, white rhinoceros (25 samples from 6 animals), and African elephants (3 samples from 2 animals) from bovine tuberculosis endemic wildlife parks and a zoo (1 elephant) within South Africa.

Species/sample type	No. of samples from known MTBC-infected animals	Conventional MGIT		Novel TiKa		Differential TTP
		MTBC recovery	MTBC median TTP (d)	MTBC recovery	MTBC median TTP (d)	Median MGIT (TTP) – Median TiKa (TTP) (d)
African buffaloes						
Tissue	26	10/26†	25.1§	23/26‡	10.8||	13.4
White rhinoceros						
Tissue	25	12/25†	9§	25/25‡	6.9||	3.9
African elephants						
Tissue	3	2/3	10.5	3/3	4	6.5
BALF	1	1/1	16.1	1/1	7.5	8.6
ETT	1	0/1	ND	1/1	10	ND
TW	1	0/1	ND	1/1	4.3	ND

Culture positive refers to measurable growth in the *Mycobacterium* growth indicator tube (MGIT) detected by the Bactec MGIT 960 mycobacterial detection system (unconfirmed MTBC). Animals were confirmed as MTBC (either *M. bovis* or *M. tuberculosis*)*-*infected by mycobacterial culture and strain typing.^28^ MTBC recovery is the ratio of MTBC confirmed (ZN stain – positive, no blood agar growth, and RD-PCR^28^ confirmed) positive cultures divided by the number of specimens cultured. Within a row, MTBC recovery rates with a different superscript (†,‡) are significantly different (*p* < 0.01). MTBC TTP = time to positivity of the MGIT tube from inserting it into the Bactec 960 machine until first MTBC growth is observed by the machine. Within a row, TTP values with a different superscript (§,||) are significantly different (*p* < 0.01). White rhinoceros tissue samples were collected opportunistically from 6 poached rhinoceros carcasses. African elephant tissue samples were collected from a known *M. tuberculosis–*infected and *M. bovis–*infected animal. Bronchoalveolar lavage fluid (BALF), trunk wash (TW), and endotracheal tube wash (ETT) samples were collected from the known *M. bovis–*infected animal. ND = not done.

Similarly, 25 tissue specimens collected from 6 *M. bovis*–infected rhinoceros (4–6 specimens per animal) were confirmed to have *M. bovis* present. Using these samples, an increased MTBC culture recovery rate of 25 of 25 was observed when using the TiKa system compared to the MGIT system (12 of 25 positive). Additionally, a significant (*p* = 0.0041) average overall reduction in TTP of 3.9 d was observed with TiKa (Table 1). Results from both systems were compared using the McNemar test and found to be statistically different (*p* = 0.0009). Both culture techniques successfully detected *M. bovis* in 12 of 25 tissue specimens, although 13 of 25 tissue specimens were MGIT-negative but TiKa-positive (Table 1).

Of the 3 tissue specimens collected from 2 MTBC (*M. tuberculosis* and *M. bovis*)-infected African elephants, MTBC was recovered from 3 of 3 using TiKa compared to 2 of 3 for MGIT (Table 1), with an overall reduction in TTP of 6.5 d using the TiKa system. Both the MGIT and TiKa systems isolated *M. bovis* and *M. tuberculosis* from the same 2 specimens, and the TiKa system detected an additional specimen with *M. bovis* (Suppl. Table 2). From the single TW specimen, 1 BALF specimen, and 1 endoscope tube wash collected from the *M. tuberculosis*–infected elephant, TiKa culture detected *M. tuberculosis* in all 3, compared to only 1 in MGIT, which was the BALF sample (Table 1; Suppl. Table 2). A reduction in TTP of 8.6 d were observed with TiKa for the BALF specimen.

## Discussion

Our findings support previous findings and suggest that NALC-NaOH as a decontamination agent reduces mycobacterial growth and that this outcome can be avoided by the alternative use of the TiKa-KiC decontamination agent and supplement B.^3,19^ Only one study, using TiKa in clinical animal samples, has been published in which MAP was cultured from cattle lymphoid tissue.^3^ That study showed 1) a similar diagnostic sensitivity of the TiKa system to that of a qPCR, 2) increased detection of non-replicating MAP of up to 3 logs above that detected by the conventional MGIT system, and 3) direct carryover contamination by viable non-mycobacterial flora in only 1 of 470 sample preperations.^3^ To our knowledge, the TiKa system has not been reported previously for use on African wildlife postmortem tissue and antemortem respiratory clinical specimens from animals with known MTBC infections. When culturing various tissue and respiratory specimens collected from known MTBC-infected buffaloes, rhinoceros, and elephants, we observed overall enhanced mycobacterial growth when using the TiKa system compared to the conventional MGIT system. Enhanced mycobacterial growth was defined as an increase in MTBC recovery with a reduction in TTP and reduced false negativity.

Notably, for the different types of specimens collected from the confirmed MTBC-infected animals, negative culture results were observed, leading to discordant results between systems. Reasons for this may include: 1) absence of viable bacilli caused by freeze–thaw cycles of specimens, 2) differences in mycobacterial numbers between samples derived from an original paucibacillary specimen and used in parallel culture on both systems, 3) naturally low MTBC numbers present in specimens, especially antemortem respiratory samples, combined with a high concentration of fast-growing environmental non-tuberculous mycobacteria (NTM) that outcompete the slow-growing pathogenic MTBC for nutrients, masking their presence by PCR detection, and 4) the harsh decontamination procedure of the MGIT system, inhibiting growth of low numbers of any pathogenic MTBC present. Although samples harvested from the upper respiratory tract may contain high concentrations of NTM and, therefore, require harsher decontamination to prevent contamination in culture, the concentration range of NTM at which optimal decontamination can be achieved by both systems is unknown.

Our findings strongly support the use of the TiKa agents to enhance culture recovery of paucibacillary specimens, regardless of specimen type. The implications of our findings are particularly important in situations in which the diagnosis of TB in animals relies primarily on mycobacterial culture of antemortem respiratory tract samples. Our results have shown that TiKa decontamination and culture can be incorporated easily and will enhance existing routine laboratory methods. This innovation will help veterinarians and regulatory agencies make more rapid, informed decisions regarding mycobacterial disease management based on improved culture results for animals.

The limitations of our study include limited numbers and the sole use of animals from bTB endemic parks. These limitations can be overcome by conducting a thorough evaluation of the specificity of the TiKa system by using clinical specimens from larger cohorts of animals from known MTBC-negative locations, as well as increasing the number of samples from known infected individuals.

## Supplemental Material

sj-pdf-1-vdi-10.1177_10406387211044192 – Supplemental material for Improved detection of *Mycobacterium tuberculosis* and *M. bovis* in African wildlife samples using cationic peptide decontamination and mycobacterial culture supplementation Supplemental material, sj-pdf-1-vdi-10.1177_10406387211044192 for Improved detection of *Mycobacterium tuberculosis* and *M. bovis* in African wildlife samples using cationic peptide decontamination and mycobacterial culture supplementation by Wynand J. Goosen, Léanie Kleynhans, Tanya J. Kerr, Paul D. van Helden, Peter Buss, Robin M. Warren and Michele A. Miller in Journal of Veterinary Diagnostic Investigation
