# Supplementary material for: Improved detection of Mycobacterium tuberculosis and M. bovis in African wildlife samples using cationic peptide decontamination and mycobacterial culture supplementation
Source: J Vet Diagn Invest. 2021 Sep 11;34(1):61–7. doi: 10.1177/10406387211044192 (PMC8688974; doi:10.1177/10406387211044192)
Supplement: sj-pdf-1-vdi-10.1177_10406387211044192 – Supplemental material for Improved detection of Mycobacterium tuberculosis and M. bovis in African wildlife samples using cationic peptide decontamination and mycobacterial culture supplementation [file sj-pdf-1-vdi-10.1177_10406387211044192.pdf]

JVDI: Supplemental material

Goosen WJ, et al. Improved detection of *Mycobacterium tuberculosis* and *M. bovis* in African wildlife samples using cationic peptide decontamination and mycobacterial culture supplementation

Supplemental Table 1. Mycobacterial culture results for tissue samples collected from 48 African buffaloes (*Syncerus caffer*) sampled from Hluhluwe iMfolozi Park (KwaZulu Natal), a *M. bovis* endemic wildlife park in South Africa.

		Conventional Bactec 960 MGIT				Novel TiKa-MGIT			
Animal	Tissue	MGIT result	TTP (d; h)	ZN stain	RD-PCR	MGIT result	TTP (d; h)	ZN stain	RD-PCR
1	Pooled head LNs	+	20;13	+	*M. bovis*	NG	ND	ND	ND
2	Pooled thoracic LNs	+	27;03	+	*M. bovis*	+	9;18	+	*M. bovis*
3	Pooled head LNs	+	54;23	+	–	NG	ND	ND	ND
4	Pooled thoracic LNs	+	46;14	–	–	+	11;14	+	*M. bovis*
5	Prescapular LN lesion	+	24;18	+	–	+	8	+	*M. bovis*
6	Retro LN lesion	NG	ND	ND	ND	NG	ND	ND	ND
7	Pooled head LNs	NG	ND	ND	ND	NG	ND	ND	ND
8	Pooled thoracic LNs	+	14;07	–	–	+	13;04	+	*M. bovis*
9	Tracheobronchial LN lesion	+	30;01	–	–	NG	ND	ND	ND
10	Tracheobronchial LN lesion	+	24;00	–	–	+	10;17	+	*M. bovis*
11	Pooled head LNs	+	25;05	+	*M. bovis*	+	1;21	+	*M. bovis*
12	Pooled thoracic LNs	+	24;22	–	–	+	17;21	+	*M. bovis*
13	Mediastinal LN lesion	NG	ND	ND	ND	NG	ND	ND	ND
14	Lung lesion	+	15;13	–	–	+	8;22	+	*M. bovis*
15	Tracheobronchial LN lesion	+	48;04	+	*M. bovis*	+	5;16	+	*M. bovis*
16	Parotid LN lesion	+	12;19	–	–	+	11	+	*M. bovis*
17	Pooled head LNs	+	43;17	–	–	+	12;06	+	*M. bovis*
18	Pooled thoracic LNs	NG	ND	ND	ND	NG	ND	ND	ND
19	Pooled head LNs	+	13;05	–	–	+	10;18	+	*M. bovis*
20	Pooled thoracic LNs	+	10;00	+	*M. bovis*	+	3;08	+	*M. bovis*
21	Pooled thoracic LNs	NG	ND	ND	ND	NG	ND	ND	ND
22	Pooled thoracic LNs	NG	ND	ND	ND	NG	ND	ND	ND
23	Prescapular LN lesion	+	38;19	+	*M. bovis*	NG	ND	ND	ND
24	Pooled thoracic LNs	NG	ND	ND	ND	NG	ND	ND	ND

25	Retro LN lesion	+	32;08	–	–	+	14;03	+	*M. bovis*
26	Pooled thoracic LNs	+	38;01	–	–	+	10;19	+	*M. bovis*
27	Pooled head LNs	NG	ND	ND	ND	NG	ND	ND	ND
28	Pooled thoracic LNs	+	16;09	+	*M. bovis*	NG	ND	ND	ND
29	Pooled head LNs	+	44;16	+	*M. bovis*	+	17;07	+	*M. bovis*
30	Pooled thoracic LNs	NG	ND	ND	ND	NG	ND	ND	ND
31	Pooled head LNs	+	4;13	–	–	+	12;01	+	*M. bovis*
32	Pooled thoracic LNs	NG	ND	ND	ND	NG	ND	ND	ND
33	Mediastinal LN lesion	+	6;07	+	*M. bovis*	+	6;16	+	*M. bovis*
34	Pooled head LNs	NG	ND	ND	ND	+	10;14	+	*M. bovis*
35	Pooled thoracic LNs	+	30;05	–	–	NG	ND	ND	ND
36	Pooled thoracic LNs	+	40;01	–	–	NG	ND	ND	ND
37	Pooled head LNs	+	44	–	–	NG	ND	ND	ND
38	Pooled head LNs	+	12	–	–	NG	ND	ND	ND
39	Tonsil LN lesion	+	46;00	–	–	+	10;11	+	*M. bovis*
40	Pooled thoracic LNs	+	46	–	–	NG	ND	ND	ND
41	Pooled thoracic LNs	+	49;02	–	–	NG	ND	ND	ND
42	Pooled thoracic LNs	+	30;08	–	–	+	32;22	+	*M. bovis*
43	Pooled thoracic LNs	+	18;09	–	–	NG	ND	ND	ND
44	Pooled thoracic LNs	+	13;09	+	–	NG	ND	ND	ND
45	Pooled thoracic LNs	+	48;09	+	–	NG	ND	ND	ND
46	Pooled thoracic LNs	NG	ND	ND	ND	NG	ND	ND	ND
47	Pooled thoracic LNs	NG	ND	ND	ND	+	18;01	+	*M. bovis*
48	Pooled thoracic LNs	+	25;09	+	*M. bovis*	+	6;01	+	*M. bovis*

Tissue samples, including head, lung, and peripheral lymph nodes (LNs), were collected from 48 African buffaloes. All samples from each animal were pooled for downstream mycobacterial culture using the Bactec 960 MGIT (Becton Dickinson) and novel TiKa-MGIT (TiKa Diagnostics) method. ND = not done; NG = no growth; RD-PCR = region of difference PCR; TTP = time to positivity; ZN = Ziehl–Neelsen staining; + = positive; – = negative.

Supplemental Table 2. Mycobacterial culture results for tissue and respiratory samples with known mycobacterial culture results from 12 white rhinoceros (*Ceratotherium simum*) and 22 African elephants (*Loxodonta africana*) sampled from Kruger National Park, a *M. bovis* endemic wildlife park in South Africa.

			Conventional MGIT culturing				Novel TiKa-MGIT			
Animal	Species	Sample	MGIT result	TTP (d; hr)	ZN stain	RD-PCR	MGIT result	TTP (d; hr)	ZN stain	RD-PCR
1	WR	BALF	NG	ND	ND	ND	NG	ND	ND	ND
1	WR	Tracheobronchial LN lesion	NG	ND	ND	ND	+	8;04	SUS	*M. bovis*
1	WR	Peripheral LN	NG	ND	ND	ND	+	6;11	+	*M. bovis*
1	WR	Prescapular LN lesion	NG	ND	ND	ND	NG	ND	ND	ND
1	WR	Pooled head LNs	NG	ND	ND	ND	+	7;14	+	*M. bovis*
1	WR	Lung LNs	NG	ND	ND	ND	+	7;20	+	*M. bovis*
1	WR	Retropharyngeal LN	NG	ND	ND	ND	+	14;18	+	*M. bovis*
2	WR	Abdominal LNs	+	32;00	–	–	NG	ND	ND	ND
2	WR	Lung lesion	+	33;00	–	–	+	4;01	+	*M. bovis*
2	WR	Peripheral LN	+	56;00	–	–	NG	ND	ND	ND
2	WR	Thoracic LN	NG	ND	ND	ND	+	3;23	+	*M. bovis*
3	WR	Head LN	+	13;12	+	*M. bovis*	+	13;13	+	*M. bovis*
3	WR	Abdominal LN	+	11;20	+	*M. bovis*	+	6;0	+	*M. bovis*
3	WR	Peripheral LN	+	19;13	+	*M. bovis*	+	4;7	+	*M. bovis*
3	WR	Tracheobronchial LN lesion	+	8;15	+	*M. bovis*	+	7;9	+	*M. bovis*
3	WR	Lung lesion	+	9;21	+	*M. bovis*	+	4;5	+	*M. bovis*
3	WR	Mammary gland LN lesion	+	27;21	SUS	–	+	4;7	+	*M. bovis*
4	WR	Head LNs	NG	ND	ND	ND	+	5;12	+	*M. bovis*
4	WR	Thoracic LNs	+	7;5	–	–	+	18;10	+	*M. bovis*
4	WR	Peripheral LNs	NG	ND	ND	ND	+	4;13	+	*M. bovis*
4	WR	Lung tissue	+	15;13	+	*M. bovis*	+	7;21	+	*M. bovis*
5	WR	Lung tissue	+	4;15	+	*M. bovis*	+	9;18	+	*M. bovis*
5	WR	Head LNs	+	2;15	+	*M. bovis*	+	4;7	+	*M. bovis*

5	WR	Abdominal LN	+	7;2	–	–	NG	ND	ND	ND
5	WR	Peripheral LN	+	8;23	+	*M. bovis*	+	11;9	+	*M. bovis*
5	WR	Thoracic LN	+	7;04	–	*M. bovis*	+	9;6	+	*M. bovis*
6	WR	Head LN	+	53;3	–	–	+	7;9	+	*M. bovis*
6	WR	Thoracic LN	+	8;15	+	*M. bovis*	+	3;0	+	*M. bovis*
6	WR	Lung lesion	+	33;19	+	*M. bovis*	+	5;12	+	*M. bovis*
6	WR	Peripheral LN	+	9;8	–	–	+	8;14	+	*M. bovis*
7	WR	BALF	+	0;19	SUS	–	+	4;12	–	–
7	WR	BALF	+	7;0	SUS	–	NG	ND	ND	ND
7	WR	BALF	+	21;3	–	–	NG	ND	ND	ND
7	WR	BALF	+	16;9	–	–	NG	ND	ND	ND
7	WR	BALF	NG	ND	ND	ND	NG	ND	ND	ND
7	WR	BALF	NG	ND	ND	ND	NG	ND	ND	ND
7	WR	BALF	NG	ND	ND	ND	NG	ND	ND	ND
8	WR	BALF	+	0;22	SUS	–	NG	ND	ND	ND
8	WR	BALF	+	4;9	SUS	–	NG	ND	ND	ND
8	WR	BALF	+	25;10	–	–	NG	ND	ND	ND
8	WR	BALF	+	34;21	–	–	NG	ND	ND	ND
8	WR	BALF	NG	ND	ND	ND	NG	ND	ND	ND
8	WR	BALF	+	26;12	+	–	+	19;2	–	–
8	WR	BALF	NG	ND	ND	ND	NG	ND	ND	ND
9	WR	BALF	+	4;9	SUS	–	NG	ND	ND	ND
9	WR	BALF	+	29;23	–	–	NG	ND	ND	ND
9	WR	BALF	+	10;22	SUS	–	NG	ND	ND	ND
9	WR	BALF	+	3;3	–	–	NG	ND	ND	ND
9	WR	BALF	NG	ND	ND	ND	NG	ND	ND	ND
9	WR	BALF	+	6;9	SUS	–	NG	ND	ND	ND
9	WR	BALF	NG	ND	ND	ND	+	11;19	–	–
9	WR	BALF	+	2;11	–	–	+	9;22	–	–
10	WR	BALF	+	2;23	SUS	–	NG	ND	ND	ND
10	WR	BALF	+	6;17	SUS	–	NG	ND	ND	ND
10	WR	BALF	+	9;8	–	–	NG	ND	ND	ND

10	WR	BALF	NG	ND	ND	ND	+	4;16	–	–
10	WR	BALF	NG	ND	ND	ND	NG	ND	ND	ND
10	WR	BALF	+	43,4	SUS	–	NG	ND	ND	ND
10	WR	BALF	NG	ND	ND	ND	NG	ND	ND	ND
11	WR	BALF	NG	ND	ND	ND	+	13;20	–	–
12	WR	BALF	+	5;11	SUS	–	NG	ND	ND	ND
1	AE	TW	+	7;11	+	–	+	4;08	–	*M. tuberculosis*
1	AE	BALF	+	19;14	+	*M. tuberculosis*	+	5;11	–	*M. tuberculosis*
1	AE	ETT	+	12;11	+	–	+	9;23	+	*M. tuberculosis*
1	AE	Lung tissue	+	6;10	+	*M. tuberculosis*	+	3;23	+	*M. tuberculosis*
2	AE	Head LN	NG	ND	ND	ND	+	2;21	–	*M. bovis*
2	AE	Abdominal LNs	NG	ND	ND	ND	NG	ND	ND	ND
2	AE	Thoracic LNs	+	14;12	+	*M. bovis*	+	3;23	–	*M. bovis*
2	AE	Peripheral LNs	NG	ND	ND	ND	NG	ND	ND	ND
2	AE	Lung tissue	+	15;14	+	–	NG	ND	ND	ND
3	AE	Thoracic LNs	+	15;22	–	–	+	2;13	–	–
3	AE	Lung	+	10;0	–	–	+	1;14	–	–
4	AE	BALF	+	2;19	–	–	ND	ND	ND	ND
5	AE	BALF	+	14;0	–	–	NG	ND	ND	ND
6	AE	BALF	NG	ND	ND	ND	NG	ND	ND	ND
7	AE	BALF	+	8;19	–	–	NG	ND	ND	ND
8	AE	BALF	+	3;14	–	–	+	20;11	–	–
9	AE	BALF	+	5;11	–	–	NG	ND	ND	ND
10	AE	BALF	+	7;22	–	–	NG	ND	ND	ND
11	AE	BALF	+	3;20	–	–	NG	ND	ND	ND
12	AE	BALF	NG	ND	ND	ND	NG	ND	ND	ND
13	AE	BALF	+	13;6	–	–	NG	ND	ND	ND
14	AE	BALF	+	6;09	–	–	NG	ND	ND	ND
15	AE	BALF	NG	ND	ND	ND	NG	ND	ND	ND
16	AE	BALF	NG	ND	ND	ND	NG	ND	ND	ND
17	AE	BALF	NG	ND	ND	ND	NG	ND	ND	ND
18	AE	BALF	NG	ND	ND	ND	NG	ND	ND	ND

19	AE	BALF	+	8;13	–	–	NG	ND	ND	ND
20	AE	BALF	NG	ND	ND	ND	NG	ND	ND	ND
21	AE	BALF	NG	ND	ND	ND	NG	ND	ND	ND
22	AE	BALF	+	11;15	–	–	NG	ND	ND	ND
5	AE	TW	+	15;10	–	–	+	3;06	–	–
6	AE	TW	+	29;10	–	–	NG	ND	ND	ND
8	AE	TW	+	5;14	–	–	+	6;20	–	–
10	AE	TW	NG	ND	ND	ND	NG	ND	ND	ND
12	AE	TW	+	8;18	–	–	NG	ND	ND	ND
13	AE	TW	+	18;0	–	–	NG	ND	ND	ND
14	AE	TW	+	11;22	–	–	NG	ND	ND	ND
15	AE	TW	+	7;17	–	–	NG	ND	ND	ND
16	AE	TW	NG	ND	ND	ND	NG	ND	ND	ND
17	AE	TW	NG	ND	ND	ND	NG	ND	ND	ND
18	AE	TW	+	3;04	–	–	NG	ND	ND	ND
19	AE	TW	+	1;0	–	–	NG	ND	ND	ND
20	AE	TW	+	2;06	–	–	NG	ND	ND	ND
21	AE	TW	NG	ND	ND	ND	NG	ND	ND	ND
22	AE	TW	+	2; 15	–	–	NG	ND	ND	ND

Tissue samples including head, lung, and peripheral lymph nodes (LNs) and respiratory samples like bronchoalveolar lavage fluid (BALF), trunk washes (TW) and endotracheal tube washes (ETT) were collected from white rhinoceros (WR) and African elephants (AE). Samples from each animal were cultured using the Bactec 960 MGIT (Becton Dickinson) and novel TiKa-MGIT (TiKA Diagnostics) method. ND = not done; NG = no growth; RD-PCR = region of difference PCR; TTP = time to positivity; SUS = suspect; ZN = Ziehl–Neelsen staining; + = positive; – = negative.
